# Identification of Factors Predicting False-Negative Results in Sentinel Lymph Node Biopsy in Patients Undergoing Surgery for Breast Cancer: A Single-Center Retrospective Study

**DOI:** 10.7759/cureus.85891

**Published:** 2025-06-12

**Authors:** Sümeyra Bölük, Salih Bölük

**Affiliations:** 1 General Surgery, İstanbul Sultan Abdülhamid Han Eğitim ve Araştırma Hastanesi, Istanbul, TUR; 2 General Surgery, Gebze Medical Park Hospital, Istanbul, TUR

**Keywords:** breast cancer, false negative, oncology, sentinel lymph node, surgery

## Abstract

Background

In breast cancer surgery, sentinel lymph node biopsy (SLNB) is routinely utilized for axillary assessment. Frozen section examination of the sentinel lymph node (SLN) is used to guide the decision for axillary dissection. Adjuvant treatment is also planned based on the final pathological examination of the axilla and the mastectomy/breast-conserving surgery specimen. In rare cases, even when the frozen section examination of the SLN is negative, micrometastases and macrometastases can still be detected in the final pathologic examination. In our study, we aimed to analyze the characteristics of patients who underwent surgery for breast cancer and were found to have false-negative results in SLNB. We aimed to identify potential predictive markers for false-negative results in SLNB.

Methodology

A total of 206 patients with breast cancer who underwent surgery in our department between January 2018 and September 2023 were evaluated retrospectively. In total, 12 patients with false-negative SLNB results and 12 patients with true-negative SLNB results were reviewed. Demographic information of the patients, type of breast malignancy, hormone receptor status, the number of lymph nodes dissected in SLN sampling, and whether the patients received neoadjuvant treatment were recorded.

Results

The results of 12 cases with false-negative results in SLNB were compared with 12 cases with true-negative results. The ages of the cases ranged from 33 to 80 years, and the mean age was 57.00 ± 12.55 years. The mean age was 59.92 ± 9.72 years in the group with false-negative SLNB results and 54.08 ± 14.71 in the group with true-negative SLNB results. No significant difference was found between the groups (p > 0.05). No statistically significant difference was determined in tumor size, stage, estrogen receptor, progesterone receptor (PR), C-ERB, HER-2, and E-cadherin between the groups (p > 0.05). It is noteworthy that PR was detected at a higher rate in the false-negative SLNB group. In cases with false-negative results in SLNB, the absence of treatment response in the postoperative pathological examination was found to be statistically significantly higher. On the other hand, complete response and partial response rates were significantly higher in the SLNB true-negative group (p = 0.011 and p < 0.05). However, because response rates cannot be assessed preoperatively, they cannot be considered a predictive factor. Mean Ki-67 (%) of the cases with true-negative SLNB results was statistically significantly higher than the false-negative group (p = 0.017 and p < 0.05).

Conclusions

SLNB is routinely performed in breast cancer for the evaluation of the axilla. Examining a single blue-stained lymph node may be sufficient for SLN assessment. In the preoperative period, there is no imaging method, pathological finding, or data that can definitively predict the probability of a positive SLN. Even when the patient has received neoadjuvant therapy, unnecessary lymph node dissection should be avoided during SLN sampling.

## Introduction

Breast cancer is the most common cancer in women. According to mortality statistics, it is the second leading cause of cancer-related deaths [1,2]. Early diagnosis and treatment are crucial. Today, increased accessibility to breast cancer screening has led to higher rates of early-stage breast cancer detection [3]. After diagnosis, a treatment plan must be promptly established.

Surgical options for breast cancer have evolved over time from radical mastectomy to modified radical mastectomy and breast-conserving surgery. Similarly, axillary surgery has changed from routine level 2 lymph node dissection to sentinel lymph node biopsy (SLNB) [4]. Some recent studies have suggested that SLNB does not contribute to prognosis in selected cases [5]. For patients with a negative SLNB, axillary dissection is avoided. While frozen section examination offers preliminary information, false negatives are possible, though they are uncommon. For sentinel lymph node (SLN) sampling, a blue-stained lymph node may be sufficient. In our study, we aimed to identify factors that may contribute to false-negative SLNB results.

## Materials and methods

Study design

Patients diagnosed with breast cancer who underwent surgery in our hospital between January 2018 and September 2023 were reviewed. In total, the results of 206 breast cancer patients were analyzed. Overall, 12 patients whose SLNB was evaluated as false negative were included in the study.

Study population and sample size

In total, 6 (50%) of the 12 patients had received neoadjuvant treatment, and 6 (50%) had not. A total of 12 patients whose SLNB was true negative were selected randomly as the comparison group. Of these 12 patients, 6 (50%) had also received neoadjuvant treatment, and 6 (50%) did not.

Study measures

The patients’ demographic characteristics, pathology data, use of neoadjuvant therapy, and PET-CT results, if available, were recorded and reviewed. We aimed to determine whether there was a factor that could predict false-negative SLNB results.

Statistical analysis

SPSS version 27 (IBM Corp., Armonk, NY, USA) was used for statistical analysis. Quantitative variables are presented as mean, standard deviation, median, minimum, and maximum values, whereas descriptive statistical methods such as frequency and percentage were used for qualitative variables. The Shapiro-Wilk test and box plot graphics were employed to evaluate the suitability of the data for normal distribution. The Mann-Whitney U test was used to assess the variables that did not follow a normal distribution between the two groups. Pearson’s chi-squared test, Fisher’s exact test, and Fisher-Freeman-Halton test were utilized to compare qualitative data. The results were evaluated with a 95% confidence interval, and significance was set at p-values <0.05.

Ethics considerations

Ethics committee approval was obtained from the non-interventional ethics committee of Kocaeli Health and Technology University (approval number: 2025-138; dated: 10.03.2025).

## Results

The study was conducted among 206 patients diagnosed with breast cancer who underwent surgery in our hospital between January 2018 and September 2023. The results of 12 cases with false-negative results in SLNB were compared with those of 12 cases with true-negative results in SLNB. Age and gender were evaluated as sociodemographic characteristics. All 24 patients were women. The ages of the cases ranged from 33 to 80 years, with the mean age being 57.00 ± 12.55 years. The mean age of the group with false-negative SLNB results was 59.92 ± 9.72 years compared to 54.08 ± 14.71 on the group with true-negative SLNB results. No significant difference was observed between the two groups (p > 0.05). One patient’s mass was too small to be biopsied (when evaluated by interventional radiology). When evaluated together with radiology, it was decided to resect the mass with SLN sampling because of the high probability of malignancy. Due to the size of the mass, the diagnosis was not made by frozen section examination (Table 1). Results of preoperative mass biopsy, axillary lymph node biopsy, and axillary evaluation on PET-CT did not show statistically significant differences between groups (p > 0.05) (Table 1).

**Table 1:** Comparison of tumor biopsy, axillary LN biopsy, and PET-CT results by groups. ^a^: Fisher-Freeman-Halton test; ^b^: Fisher’s exact test; ^c^: Pearson’s chi-square test. SLNB: sentinel lymph node biopsy; LN: lymph node; PET-CT: positron emission tomography-computed tomography

		Group		P-value
		SLNB false negative (n = 12), n (%)	SLNB true negative (n = 12), n (%)	
Breast mass biopsy result	Biopsy could not be performed (small mass)	1 (8.3)	0 (0)	0.306^a^
	Invasive ductal carcinoma	3 (25)	7 (58.3)	
	Invasive lobular carcinoma	3 (25)	1 (8.3)	
	Invasive breast carcinoma	4 (33.3)	4 (33.3)	
	Invasive ductal carcinoma with neuroendocrine features	1 (8.3)	0 (0)	
Axillary biopsy	Not performed	10 (83.3)	7 (58.3)	0.371^b^
	Performed	2 (16.7)	5 (41.7)	
Axillary involvement in PET-CT	Negative	9 (75)	12 (100)	0.212^c^
	No PET-CT	2 (16.7)	0 (0)	
	Suspicious positive	1 (8.3)	0 (0)	

A statistically significant difference in treatment response was found among patients who received neoadjuvant therapy when compared across the groups (p = 0.011 and p < 0.05). In cases with false-negative results in SLNB, the absence of treatment response in the postoperative pathological examination was found to be statistically significantly higher. On the other hand, the rates of complete and partial response were significantly higher in the true-negative SLNB group (Table 2).

**Table 2:** Comparison of neoadjuvant treatment evaluations by groups. ^a^: Pearson’s chi-square test; ^b^: Fisher-Freeman-Halton test; *: p-value <0.05. SLNB: sentinel lymph node biopsy

		Group		P-value
		SLNB false negative (n = 12), n (%)	SLNB true negative (n = 12), n (%)	
Neoadjuvant therapy	No	6 (50.0)	6 (50.0)	1.000^a^
	Yes	6 (50.0)	6 (50.0)	
Pathological response to treatment in neoadjuvant treatment recipients	Response (partial)	2 (33.3)	2 (33.3)	0.011^b^ *
	No response	4 (66.6)	0 (0.0)	
	Complete response (no residual tumor)	0 (0.0)	4 (66.6)	

No statistically significant difference was found in postoperative pathology between groups (p > 0.05) (Table 3).

**Table 3:** Comparison of postoperative pathology results by groups. ^a^: Fisher-Freeman-Halton test. SLNB: sentinel lymph node biopsy

		Group		P-value
		SLNB false negative (n = 12), n (%)	SLNB true negative (n = 12), n (%)	
Postoperative pathology results	Invasive ductal carcinoma	7 (58.3)	6 (50.0)	0.282^a^
	Invasive lobular carcinoma	3 (25.0)	1 (8.3)	
	Invasive breast carcinoma	1 (8.3)	1 (8.3)	
	Invasive ductal carcinoma with neuroendocrine features	1 (8.3)	0 (0.0)	
	Mucinous carcinoma	0 (0.0)	1 (8.3)	
	Complete response (no tumor)	0 (0.0)	4 (25.1)	

Tumor size, T stage, estrogen receptor (ER), progesterone receptor (PR), C-ERB, HER-2, and E-cadherin did not show statistically significant differences between the groups (p > 0.05). Of note, PR was detected at a higher rate in the SLNB false-negative group (Table 4).

**Table 4:** Comparison of tumor-related characteristics by groups. ^a^: Mann-Whitney U test; ^b^: Fisher-Freeman-Halton test; ^c^: Fisher exact test; ^d^: Pearson’s chi-square test. SLNB: sentinel lymph node biopsy; ER: estrogen receptor; PR: progesterone receptor

		Group		P-value
		SLNB false negative (n = 12), n (%)	SLNB true negative (n = 12), n (%)	
Tumor size (cm)	Mean ± SD	2.20 ± 1.20	3.10 ± 1.10	0.089^a^
	Median (minimum-maximum)	2.3 (0.3-5)	3.4 (1.3-5)	
Classification according to tumor size	T1	6 (50.0)	3 (25)	0.403^b^
	T2	6 (50.0)	8 (66.7)	
	T3	0 (0.0)	1 (8.3)	
ER	Negative	1 (8.3)	3 (25)	0.273^d^
	Positive	11 (91.7)	9 (75.0)	
PR	Negative	1 (8.3)	5 (41.7)	0.060^c^
	Positive	11 (91.7)	7 (58.3)	
HER-2	Not assessed	9 (75)	10 (83.3)	1.000^c^
	Negative	3 (25)	2 (16.7)	
E-cadherin	Negative	6 (50)	5 (41.7)	1.000^c^
	Positive	6 (50)	7 (58.3)	

The tumor grades showed a statistically significant difference between the groups (p = 0.037 and p < 0.05). While the rates of Grade 1 and 2 were high in the SLNB false-negative group, the rates of Unspecified Grade (due to complete response to treatment) and Grade 4 were high in the SLNB true-negative group. There was no statistically significant difference between the groups in terms of lymphovascular invasion and the number of postoperative axillary lymph nodes excised (p > 0.05) (Table 5).

**Table 5:** Distributions of tumor-related pathological features. ^a^: Fisher-Freeman-Halton test; ^b^: Mann-Whitney U test; *: p-value <0.05. SLNB: sentinel lymph node biopsy

		Group		P-value
		SLNB false negative (n = 12), n (%)	SLNB true negative (n = 12), n (%)	
Grade	Grade not specified (complete response to treatment)	0 (0)	4 (33.3)	^a^0.037*
	Grade 1	3 (25)	0 (0)	
	Grade 2	6 (50)	3 (25)	
	Grade 3	3 (25)	5 (41.7)	
Lymphovascular invasion	Yes	4 (33.3)	8 (66.7)	^a^0.102
	No	8 (66.7)	4 (33.3)	
Number of dissected lymph nodes	Mean ± SD	5.25 ± 3.74	6.08 ± 6.15	^b^0.977
	Median (minimum-maximum)	4 (1-10)	4 (1-22)	

The mean Ki-67 (%) of the cases with true-negative SLNB results was statistically significantly higher than the false-negative group (p = 0.017 and p < 0.05) (Table 6).

**Table 6:** Comparison of Ki-67(%) according to SLNB results. ^a^: Mann-Whitney U test; *: p-value <0.05. SLNB: sentinel lymph node biopsy

		SLNB findings		P-value
		SLNB false negative (n = 12), n (%)	SLNB true negative (n = 12), n (%)	
Ki-67 (%)	Mean ± SD	28.17 ± 22.42	38.17 ± 19.22	^a^0.017*
	Median (minimum-maximum)	26 (2-90)	42 (10-80)	

## Discussion

Breast cancer currently affects approximately one in eight women. It is the second leading cause of cancer-related mortality in women, following lung cancer. The lifetime risk of mortality from breast cancer is approximately 2.6% [6]. Early diagnosis contributes to a good prognosis. Screening programs performed starting at the age of 40 enable the early detection and treatment of breast cancer [7]. After the diagnosis, the clinical stage is determined with the help of imaging methods, and a treatment plan is created accordingly. While surgery is prioritized in early-stage breast cancers, neoadjuvant treatment comes to the fore in locally advanced or advanced-stage breast cancers. The appropriate treatment plan for patients is created in a multidisciplinary manner [8].

In early-stage breast cancer, axillary lymph node involvement is one of the key factors that determines the type of adjuvant therapy and prognosis after surgery [9,10]. Although imaging methods provide preliminary information about the status of the axilla, their sensitivity is not as high as that of SLNB frozen section examination. [11]. Hence, perioperative SLNB frozen section examination is planned for patients who are primarily scheduled for surgery [12]. In our study, SLNB was performed on 185 of the 206 breast cancer patients who underwent surgery, while 21 patients did not undergo SLNB. The reasons for not performing SLNB included the surgeon’s preference for a level 2 axillary dissection (removal of level 1 and 2 lymph nodes) and the indication for palliative surgery.

In cases of a positive SLN, it is typically recommended to perform axillary dissection, according to standard practice. However, studies have also shown that axillary dissection in the case of a positive SLNB does not contribute to prognosis, even if the patient has received neoadjuvant therapy for axillary lymph node positivity. In this case, a minimally invasive approach to the axilla comes to the fore. It has also been stated that SLNB may not be performed in selected cases [13]. Therefore, minimum dissection should be targeted considering postoperative morbidity. In our study, an analysis of the 21 patients who did not undergo SLNB revealed that the decision not to perform the procedure was due to various reasons. Significant difference was not found between the number of dissected lymph nodes between the groups (false-negative and true-negative SLNB).

SLN false negativity occurs at a rate of 5-10% [14]. This rate was found to be 5.8% in our study, which is consistent with the literature. For SLNB, evaluation of a single lymph node stained with methylene blue or detected by other methods is sufficient. In the earlier literature, studies primarily focused on the number of lymph nodes that needed to be excised for adequate axillary evaluation. Over time, research has increasingly emphasized targeted axillary surgery, aiming to reduce the number of lymph nodes excised [15,16]. In our study, the average number of lymph nodes dissected from 185 patients who underwent SLNB was determined to be 5.6 (minimum-maximum: 1-7). It has also been observed that the number of lymph nodes dissected for SLNB tends to decrease over the years. A false-negative result in SLNB does not necessarily imply that the patient will require additional axillary dissection [17]. In our study, micrometastases were detected in two patients. Macrometastases were detected in the other 10 patients, and none of the 12 patients with false-negative SLNB results underwent additional axillary surgery.

Although a false-negative result in SLNB does not always require a repeat axillary dissection, our study aimed to identify potential factors that could predict such an outcome. Upon reviewing all the data, the Ki-67 % rate was significantly higher in the true-negative SLNB group. However, considering that Ki-67% elevation is a poor prognostic factor, we do not think that it can be used to predict false-negative SLNB. In addition, considering that the tumor grade is determined by postoperative pathological examination, the significant result obtained regarding the tumor grade does not contribute to the prediction of false negativity. No statistically significant difference was found in the evaluation of other variables such as axillary LN biopsy, PET-CT results, T stage, ER, PR, C-ERB, HER-2, E-cadherin positivity/negativity, lymphovascular invasion status, and the number of lymph nodes dissected from the axilla (p > 0.05).

Study limitations

Study limitations include the number of patients, the random selection of the control group, and the possible result differences. The details of the neoadjuvant treatment received by the patients were not provided in the context of the study.

## Conclusions

SLNB should always be prioritized in the approach to the axilla in breast cancer, even if the patient has received neoadjuvant treatment (in treatment-responsive groups). Of note, the number of lymph nodes removed in SLNB does not contribute to the determination of positivity, and the false-negative rate is low. In this case, dissection of a lymph node detected by various methods (staining, marking, nuclear methods) may be sufficient. It is important to avoid dissecting a larger number of lymph nodes solely based on the concern of a potential false-negative result in SLNB.
